# Factor influencing women with learning disabilities deciding to, and accessing, cervical and breast cancer screening: Findings from a Q methodology study of women with learning disabilities, family and paid carers

**DOI:** 10.1111/ecc.13702

**Published:** 2022-09-12

**Authors:** Kate Sykes, Grant J. McGeechan, Hannah Crawford, Emma L. Giles

**Affiliations:** ^1^ Northumbria University Newcastle upon Tyne UK; ^2^ School of Health and Life Sciences Teesside University Middlesbrough UK; ^3^ School of Social Sciences, Humanities and Law Teesside University Middlesbrough UK; ^4^ Tees, Esk and Wear Valleys NHS Foundation Trust Middlesbrough UK

**Keywords:** breast, cervical, learning disabilities, Q methodology, screening

## Abstract

**Objectives:**

To understand knowledge of, attitudes towards and decision‐making around cervical and breast cancer screening in women with learning disabilities, family carers and paid carers.

**Methods:**

A Q methodology study involving 13 women with learning disabilities, three family carers and five paid care workers, from the North‐East of England. A Q‐sort of 28 statements was completed with all participants completing a post‐Q‐sort interview to understand the reason behind the card placements. Factor analysis was completed using PQMethod and interpreted using framework analysis.

**Results:**

Factor 1, named ‘Personal choice and ownership’, explores how women with learning disabilities want to be supported to make their own decision to attend cancer screening and explored their preferred support needs. Factor 2, named ‘Protecting vs. enablement’, portrayed the battle family carers and paid care workers felt to protect women with learning disabilities from harm, whilst feeling that they were supporting women with learning disabilities to decide to attend cancer screening. Eight consensus statements were identified indicating a shared perspective.

**Conclusions:**

Cancer screening services should ensure that women with learning disabilities are supported to make informed decisions to attend cancer screening and then be further supported throughout the cancer pathway.

## 1 INTRODUCTION

In the United Kingdom, a learning disability can be broadly defined as those who struggle with learning new skills, understanding complex or new information and coping independently (Department of Health, 2001). Having a learning disability can mean that the individual faces multiple health inequalities, which can put the person at risk of disease and premature death (Emerson & Baines, 2011). There are three screening programmes, in the United Kingdom, aiming to detect the early presence of cancer of the cervix, breast and colon (Cancer Research UK, 2018). Statistics published by NHS Digital (shown in Supporting Information S1) show that cancer screening uptake is vastly different when comparing people with learning disabilities and those without learning disabilities (NHS Digital, 2020), with the largest difference in uptake being for cervical and breast cancer screening (NHS Digital, 2020).

Research has highlighted multiple barriers to decision‐making and access to cervical and breast cancer screening by women with learning disabilities (WwLD), including mobility issues, communication difficulties (Turner et al., 2012), fear and being embarrassed about going to screening (Connolly, 2013), family carer and paid care workers may lack understanding cancer (Tuffrey‐Wijne et al., 2013) and having poor health literacy (MENCAP, 2019). Even with these known barriers to cancer screening, uptake remains consistently lower than the general population (NHS Digital, 2020). Even with the acknowledgement of the range of barriers that WwLD face in deciding to attend and then access cervical and/or breast cancer screening, there is a need to identify the most influential factors that can facilitate or hinder WwLD. Research to date tends to involve WwLD alone (Truesdale‐Kennedy et al., 2011; Willis et al., 2008) or their carers (Hanna et al., 2011; McIlfatrick et al., 2011; Willis et al., 2015; Wyatt & Talbot, 2013), with very few accounting for all their perspectives in the same study (Levi et al., 2006). The combined perspectives on factors that influence access can then be utilised to make recommendation for policy and cancer screening practice to limit the inequalities faced by WwLD. Due to this, this research aimed to further understand the range of knowledge, attitudes towards and decision‐making around cervical and breast cancer screening in WwLD, family carers and paid carers.

## 2 METHODS

Approval to complete a Q methodology study (which is a way of systematically studying a person's beliefs and attitudes; Brown, 1993; Cross, 2005; Stenner & Rogers, 2004) was received from a university and the Health Research Authority. The following subsections explain the methods used to employ the Q methodology study.

### 2.1 Identifying a concourse and developing the Q‐set

A concourse consists of the range of possible opinions regarding the topic in question (Brown, 1993) that can be written or spoken about a topic that can be contested and debated (Smith et al., 1995). A concourse is usually identified through interviews, focus groups (Gubrium et al., 2012) and conducting literature reviews (Chee et al., 2015). For this study, the results of a systematic review (Byrnes et al., 2019) were collated with reports and websites. From this, 128 statements were identified (Supporting Information S2).

The concourse is then synthesised to develop a set of statements called a Q‐set (Watts & Stenner, 2012). The Q‐set aims to cover the full range of opinions in the concourse (Brown, 1993) and tends to range from 20 to 100 statements (Watts & Stenner, 2005). The Q‐set was developed through thematically clustering the concourse statements together, based on if they had a similar meaning. All authors (KS, ELG, GJM and HC) were involved in this process. Based on this, 28 statements were developed (Supporting Information S3). Each Q‐set statement started with ‘Women with a learning disability …’, to provide consistency (Watts & Stenner, 2012) and allowed the WwLD to think about themselves or other WwLD if they had not attended cancer screening previously.

### 2.2 Specifying the participants

Participants were identified using purposive sampling of three non‐NHS day centre providers, an NHS Foundation Trust and a GP practice in the North‐East of England. Gatekeepers in each location initially identified WwLD as meeting a set of inclusion criteria (Supporting Information S4). One co‐author (HC) acted as a gatekeeper for one site and was the only author who had any involvement in identifying eligible participants. The gatekeepers discussed the study with the WwLD, using an information sheet (written in an Easy Read format, which was developed through patient and public involvement and consultation between KS with HC). If WwLD were interested in taking part, they were either given the researcher's details or their contact information was passed onto the researcher (KS). The WwLD were also asked if they were happy for their family or paid care workers to be contacted to take part. If so, the WwLD provided their details. Family carers and paid care workers were provided with information by the researcher (KS), or information was sent to the WwLD to then pass on. Written informed consent was obtained from all participants.

### 2.3 Administering the Q‐sort and analysis

The fourth step was to administer the Q‐sort. This is the process where participants rank‐order the Q‐set, based on their preferences or feelings towards the Q‐set statements (Cross, 2005). The researcher (KS) was present during all data collection activities. To complete the Q‐sort, a specific grid was developed (Supporting Information S5). The grid ranged from −4 (representing strongly disagree) to +4 (representing strongly agree) with 28 spaces for each Q‐set statement.

Participants started by sorting the Q‐set into three piles (agree, disagree and neutral/unsure), each pile was taken in turn and the cards were sorted onto the Q‐grid to indicate the participant's level of agreement, or disagreement, with the card. The sorting process was completed under a forced‐choice condition, where participants were required to place a specific number of statements under each column of the Q‐grid. This instruction can make the sorting process a more manageable conceptual task, where participants can carefully consider all statements concerning each other (McKeown & Thomas, 2013). However, participants could place cards outside the predetermined grid structure, if they strongly felt that the remaining spaces did not reflect their views. Following the Q‐sort, each participant completed a brief post‐Q‐sort interview to explain their reasons for the card placement and to understand their experiences of screening. All Q‐sorts were audio recorded and transcribed by KS.

The final step was to analyse the Q‐sorts and interpret the output. The Q‐sorts were input onto PQMethod (Schmolck, 2021). The sorts were analysed using principal component analysis (PCA) with varimax rotation. This maximised the variance and provided the best mathematical solution or best fit (McKeown & Thomas, 1988). The correlation matrix generated is subjected to the varimax rotation to optimise the separation between factors and clarified the factor structure (Rogers, 1991). A factor array was also produced, which represents a ‘mean’ Q‐sort of the shared viewpoint of participants; this can then be interpreted (Watts & Stenner, 2005). Framework analysis was completed, so the authors could interpret the factor arrays. To complete the framework analysis, a Microsoft Excel spreadsheet was developed. The spreadsheet replicated the +4, +3, −3 and −4 columns of the factor array. Quotes from the transcripts were identified to understand the reasons behind the card placement on the grid. The researcher (KS) interpreted the meaning of the factors and discussed the interpretation in detail with co‐authors (ELG, GJM and HC) and two independent Q methodologists for sense checking.

## 3 RESULTS

**FIGURE 1:**
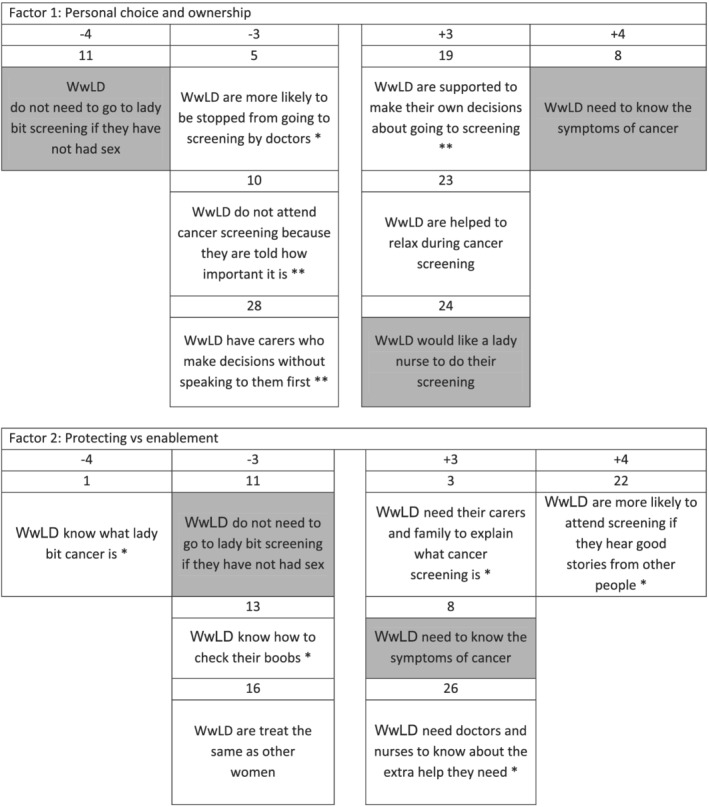
Polar ends of factor arrays. *Note*: Consensus statements are shaded; * denotes those statements, which distinguish Factor 2 from Factor 1 (at a significance level of *p* < 0.05), and ** denotes those statements, which distinguish Factor 2 from Factor 1 (at a significance level of *p* < 0.01).

A total of 13 WwLD, three family carers and five paid care workers took part (*N* = 21; 20 female, one male). Ages ranged from 20 to 69 (mean = 47.3 years). A two‐factor solution accounting for 46% of the variance and that explained the views of 19 participants was chosen (per participant factor loadings shown in Supporting Information S6). Two WwLD did not load significantly onto either factor and were classed as ‘null loaders’. Their views indicate a different and unique viewpoint to others. Factor arrays are presented in Table 1 (depicted in Supporting Information S7), with the polar ends of the factors shown in Figure 1. In the results that follow, statements will be identified in brackets by (statement number: ranking in factor array).

**TABLE 1:** Placement of each Q‐set statement on factor arrays

Item number and wording Women with learning disabilities …		Factor 1	Factor 2
1	… know what lady bit cancer is	0	−4
2	… know what boob cancer is	2	−1
3	… need their carers and family to explain what cancer screening is	0	3
4^a^	… do not attend cancer screening because they are scared	1	2
5	… are more likely to be stopped from going to screening by doctors	−3	−1
6	… are told about cancer screening by their doctor	−1	0
7	… do not always open letters so do not know about their appointment	−2	2
8^a^	… need to know the symptoms of cancer	4	3
9^a^	… speak to paid carers more than their family about their health	0	0
10	… do attend cancer screening because they are not told how important it is	−3	2
11^a^	… do not need to go to lady bits screening if they have not had sex	−4	−3
12	… are more likely to go for boob cancer screening, than lady bits cancer screening	−1	1
13	… know how to check their boobs	0	−3
14	… are helped to attend screening by talking to their doctor and nurses about what happens	0	−1
15^a^	… are asked about what would make it easier for them go to cancer screening	−2	−2
16	… are treat the same as other women	2	−3
17	… are not told about cancer screening because their carers and family are embarrassed	−2	0
18	… are told everything about the screening process, from beginning to end	−1	−2
19	… are supported to make their own decisions about going to screening	3	0
20	… are given enough time to decide if they want to be screened for cancer	1	0
21^a^	… know about what will happen in the appointment before they go	−1	−1
22	… are more likely to attend screening if they hear good stories from other people	0	4
23	… are helped to relax during cancer screening	3	0
24^a^	… would like a lady nurse to do their screening	3	1
25^a^	… find screening painful	1	1
26	… need doctors and nurses to know about the extra help they need	2	3
27	… know the reasons for cancer screening	1	−2
28	… have carers who make decisions without speaking to them first	−3	1

^a^ Consensus statements.

### 3.1 Factor 1: Personal choice and ownership

Factor 1 accounted for 28% of the total variance, with the Q‐sorts of 11 WwLD and two paid care workers defining this factor. This factor highlights a narrative of personal choice and WwLD having ownership over their healthcare decision, including whether to attend cervical and breast cancer screening or not. The importance of awareness and knowledge of cancer screening was present with participants agreeing that WwLD ‘need to know the symptoms of cancer’ (8: +4) with multiple participants strongly agreeing that it was important. However, some WwLD did not know the specific symptoms of cervical cancer: ‘it's your ovaries or the part down below, the cells and stuff’ (woman with learning disabilities 3), whereas more participants knew about breast cancer: ‘lumps in my boobs’ (woman with learning disabilities 10) ‘and bumps or things’ (woman with learning disabilities 12).

Participants felt WwLD still needed to go to cervical cancer screening if they are not or have not been sexually active (11: −4). This emphasises that WwLD's sexual status should not hinder their uptake of cervical cancer screening. A paid carer highlighted that WwLD's sexual status should not be used to determine eligibility for screening: ‘… you can't just say “you're not sexually active, you don't need to go to screening,” because you know there could be something underlying there that it could pick up’ (paid carer 01). It was also felt that WwLD know screening is an important appointment to attend (10: −3); ‘it is important to stop you getting cancer’ (woman with learning disabilities 12). Some indicated how they were made aware of its importance as ‘it's been on the News’ (woman with learning disabilities 4) and ‘the lady staff say’ (woman with learning disabilities 5). However, there was a perception that even though WwLD knew screening was important, there were other factors that influence uptake; ‘they know it is important but they won't go so what is the use’ (woman with learning disabilities 5).

It was perceived that WwLD are not stopped from going to screening by doctors (5: −3); ‘It is up to us’ (woman with learning disabilities 12). With WwLD being perceived to be ‘supported to make their own decisions about going to screening’ (19: +3) and do not ‘have carers who make decisions without speaking to them first’ (28: −3) as ‘it's up to them if they want to go or not’ (woman with learning disabilities 4), ‘I make my own decisions. I have my mind. Parent and staff haven't got my mind’ (woman with learning disabilities 12).

The need for relaxation, and how this could lead to successful screening, was acknowledged as the participants felt that WwLD ‘are helped to relax during cancer screening’ (23: +3): ‘they [screening staff] talk to you don't they, the people talk to you’ (woman with learning disabilities 4). Alongside, talking participants highlighted the importance of a female nurse completing the screening (24, +3); ‘I would feel more comfortable with a lady nurse, and not a man’ (woman with learning disabilities 3). However, it should be noted that not everyone agreed with this; for instance, one WwLD highlighted she would prefer a man to complete the screening as her doctor is male and there was an element of trust.

### 3.2 Factor 2: Protecting versus enablement

Factor 2 accounted for 18% of the total variance, with the Q‐sorts of three family carers and three paid care workers loading heavily onto this factor. The protectiveness of family carers and paid care workers to support WwLD in deciding to attend cancer screening is clear. However, this protection goes hand in hand with enablement. Family carers and paid care workers discuss multiple methods that can support WwLD. They also highlighted how changing or amending their perceptions and beliefs could greatly support WwLD to make an informed decision to attend a screening or not.

Participants that aligned to this factor perceived that WwLD need the support of paid care workers and family carers (3: +3) as well as nurses and doctors (26: +3) to support them in deciding to attend cervical and breast cancer screening or not. Specifically, WwLD rely on them to help and support them in being aware of cancer screening (3: +3). This emphasises the need for multiple parties to be involved in supporting WwLD; however, a family carer did highlight how there was a specific reliance on them as a family to support WwLD: ‘once that letter comes it's just it's up to us to explain to them’ (family carer 2). It was also noted the importance of healthcare professionals knowing about how to support and help WwLD:

Because it comes up with every aspect of somebody with learning disability's life. People need to know about the extra help they need … I mean you do have your specialist nurses like [name] but they've even tried in the past doing like little passport books that's supposed to take with them and give to somebody if they've got learning difficulties at the hospital, but they don't have time to look at them because it could be ‘wait there’ and nobody comes. (family carer 3)

This quote from a family carer also highlights how reasonable adjustments may not be effective in supporting WwLD through screening appointments, due to a lack of additional time in appointments but also lack of staffing. This again then increases the responsibility of the carers to support the WwLD.

There was a perception that WwLD were less likely to know what cervical cancer is (1: −4), know what breast cancer is (2: −1) or know how to check their breast (13: −3). These were felt to be reasons that could influence a decision to uptake cancer screening or not: ‘I don't think they will know what lady bit cancer is because I just don't think people will talk to them about it at all’ (paid carer 2).

There was a consensus that WwLD were unaware of specific aspects of cancer screening such as symptoms, yet participants highlighted WwLD do need to know what the symptoms of cancer are: ‘think it's something we should all know. We should all know what we're looking for … You know, she might not recognize the symptoms. But I certainly should’ (family carer 2). However, this carer specifically links the symptoms (and awareness of symptoms) for cervical cancer screening to sexual activity. Carers typically agreed that WwLD do need to go for cervical screening even if they have not had sexual activity (11: −3); ‘I think everybody should go for it’ (family carer 2).

Methods of improving decision‐making and experiences during cancer screening included ensuring that WwLD are told about other positive experiences of screening (22: +4); ‘if they are hearing it from their friends at day centres and things like that. It builds more of a confidence’ (paid carer 4), having doctors and nurses that know about the extra help and support the WwLD needs (26: +3); ‘they've even tried in the past doing like little passport books that's supposed to take with them and give to somebody if they've got learning difficulties at the hospital, but they don't have time to look at them’ (family carer 3) and ensuring that WwLD ‘are asked about what would make it easier for them to go to cancer screening’ (15: −2) could help WwLD decide to attend cancer screening or not and then feel supported throughout the screening process.

### 3.3 Items of consensus across Factors 1 and 2

Across both factors, there were eight consensus statements. The consensus statements will be identified in brackets by (statement number: ranking in Factor 1, ranking in Factor 2). All participants perceived that WwLD ‘do not attend cancer screening because they are scared’ (4: +1, +2); ‘need to know the symptoms of cancer’ (8: +4, +3); need to attend cervical cancer screening even when they are not sexually active (11: −4, −3); are not ‘asked about what can help them attend cancer screening’ (15: −2, −2); ‘would like a female nurse to complete their screening’ (24: +3, +1); ‘find screening painful’ (25: +1, +1) and do not ‘know about what will happen in the screening appointment before they go’ (21: −1, −1). Participants held a neutral perspective to the statement, WwLD ‘speak to paid carers more than their family about their health’ (9: 0, 0), indicating that WwLD will speak to either their family or paid carers, regarding their health.

### 3.4 Null loaders

As mentioned previously, two participants (both WwLD) were classed as ‘null loaders’. This indicated that they may hold a different perspective to the WwLD, family carers and paid care workers who load onto one of the two factors. Due to this difference in perspective, the views of both participants were not included in the final factor analysis. Some key differences between these two participants' perspectives and that of the other participants were their preference for a male to do the screening, ‘Paid carer: you would prefer a man to look at your lady bits and your boobies? Interviewee: yes’ (woman with learning disabilities 11), not needing to go to cervical cancer screening if WwLD are not sexually active, ‘Paid carer: do you think you should go for your lady bits checked? Interviewee: no’ (woman with learning disabilities 11), more unsure that WwLD would know what cervical cancer is, ‘Interviewee: some people do it depends on their needs and their learning disability they might not know about it’ (woman with learning disabilities 2), WwLD not knowing how to check their breast, ‘Researcher: Ok so do you think most women would know? Interviewee: no’ (woman with learning disabilities 2) and not feeling that WwLD are helped to relax during cancer screening, ‘Paid carer: Did you feel worked up? Interviewee: yeah Researcher: So you didn't feel chilled? I: no’ (woman with learning disabilities 2).

## 4 DISCUSSION

This study aimed to understand the knowledge of, attitudes towards and decision‐making around cervical and breast cancer screening in WwLD, family carers and paid carers. From completing a Q methodology study, two accounts were identified that reflect the attitudes and perspectives relating to WwLD accessing cervical and breast cancer screening.

Factor 1 highlighted that WwLD should have ownership over their healthcare decisions, including whether to attend cervical and breast cancer screening or not and that this was their decision to make. NHS England (2017) explained that there is a need to focus on what is important to the person within healthcare services, shifting from ‘what is the matter with you?’ towards ‘what matters to you?’ (p. 3). Specifically for WwLD, understanding what matters to them and how they can be supported to make their own decision to access cancer screening services can aid in the screening services becoming more person‐centred and flexible (based on the needs of the WwLD), as opposed to being a ‘one size fits all’ system (Kaehne, 2018).

This supports research, which also identified WwLD are fearful of the mammogram exam and find breast cancer screening painful (Arana‐Chicas et al., 2020) as well as the pain attributed to the speculum and taking of cells during cervical cancer screening (Broughton & Thomson, 2000). However, other populations of women including women with physical disabilities (Kilic et al., 2019) and women of Black African origin (Bamidele et al., 2017) have reported fearing the potential for pain, fear of the results and fear of cancer. This highlights that fear and pain are factors for women overall, which may hinder or prevent them from attending cervical and/or breast cancer screening. It is important to understand the reasons WwLD may experience fear and anxiety, especially towards cervical cancer screening, which could be due to the invasive nature of the test (Plourde et al., 2018), lack of knowledge of what screening entails and the impact potential previous sexual violence has had on the individual (McCarthy et al., 2017). This specifically reinforces the need for WwLD to attend cervical cancer screening irrespective of their sexual status. It has been documented previously that some, including healthcare professionals, may perceive WwLD should only attend cervical cancer screening if they have had sexual activity (Watts, 2008). However, WwLD should be supported to make an informed decision to attend cancer screening or not, and their sexual status should not be considered as an eligibility criterion (Lloyd & Coulson, 2014; Public Health England, 2020).

Factor 2 highlighted the protectiveness of family carers and paid care workers to support WwLD to decide to attend cancer screening. Research by Willis (2015) highlighted that family and paid carers found it difficult to explain health problems and different tests that have been completed for the person. If this is the case, supporting WwLD understand screening and may not be easy for family and paid carers (Willis, 2015). In 2019/2020, 104,723 adults with learning disabilities were living in their own homes or with their families in England (Nuffield Trust, 2021), and for those who do not live at home, various family members are typically involved in their lives and may provide care and support alongside paid carers (NHS England, 2015); this indicates the reliance and input family and paid carers could have in supporting WwLD.

### 4.1 Study limitations

Viewpoints may not be generalisable, given the small sample (Brown, 2002) and that the sample were White British. This may mean that the factors reported may not account for those of WwLD, family carers or paid carers from other cultures. In addition, only one male took part; therefore, the perspectives of males who are in relationships with WwLD or male carers are missing.

### 4.2 Clinical implications

For WwLD to make an informed decision, and then be supported to access cervical and breast cancer screening, reasonable adjustments should be identified and implemented throughout the cancer screening pathway. Proactively identifying the needs of WwLD before the invitation to attend cervical and breast cancer screening can help implement adjustments based on the WwLD needs. However, this requires WwLD to be ‘flagged’ as having learning disabilities to the screening programmes, so adjustments can be made. For instance, offering a desensitisation appointment so the WwLD is familiar with the environment and equipment. Similarly, support for family carers and paid carers to ensure their perspectives are informed by evidence, rather than personal thoughts, so they can support WwLD before they are invited to screening.

## 5 CONCLUSIONS

The findings from this study highlight multiple factors that may influence decision‐making and subsequent uptake of WwLD to cervical and breast cancer screening. This included using easy‐read documentation throughout the screening process and inviting WwLD to attend cancer screening, ensuring WwLD know the symptoms of cancer and ensuring carers are informed and supportive of WwLD decisions. Specific clinical implications have been identified, including offering reasonable adjustments throughout the cancer screening pathway from invitation through to receiving the results.

## CONFLICT OF INTEREST

None declared.

## Supporting information


**Figure S1:** Cancer screening uptake (%) for people with and without learning disabilities from 2015 to 2020
**Table S2:** Concourse statements
**Table S3:** Concourse to Q‐set statements
**Table S4:** Inclusion criteria for participants
**Figure S5:** Q‐grid
**Figure S6:** Factor arrays

## Data Availability

The data are available on request from the authors.
