# Supplementary material for: Factor influencing women with learning disabilities deciding to, and accessing, cervical and breast cancer screening: Findings from a Q methodology study of women with learning disabilities, family and paid carers
Source: Eur J Cancer Care (Engl). 2022 Sep 12;31(6):e13702. doi: 10.1111/ecc.13702 (PMC9786864; doi:10.1111/ecc.13702)

Supp 1: Cancer screening uptake (%) for people with and without learning disabilities from 2015-2020

| 1. *Cervical cancer screening uptake* |
| --- |
| 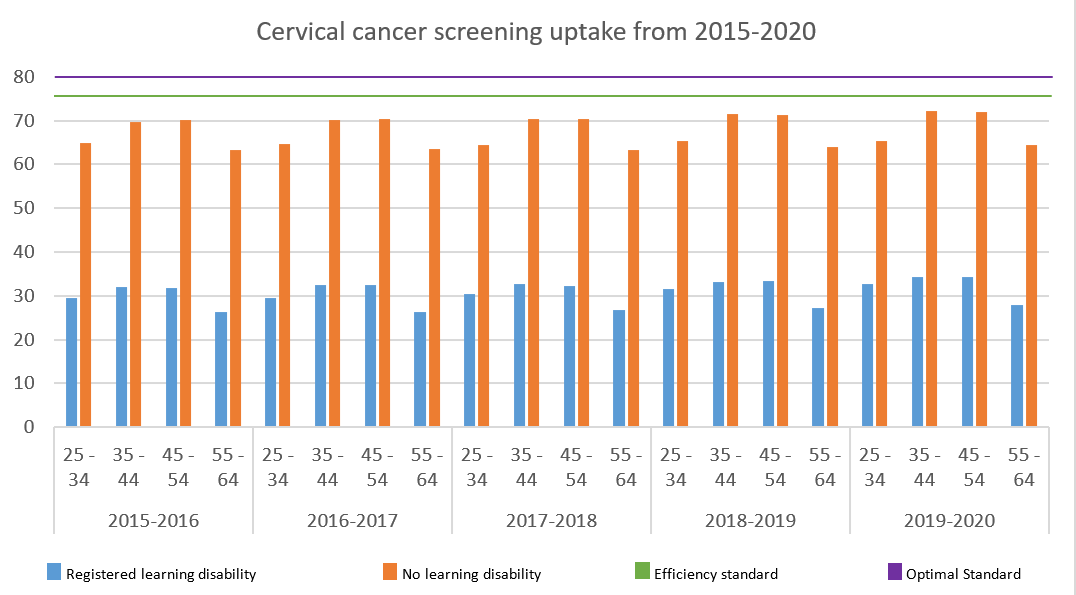 |
| 1. *Breast cancer screening uptake* |
| 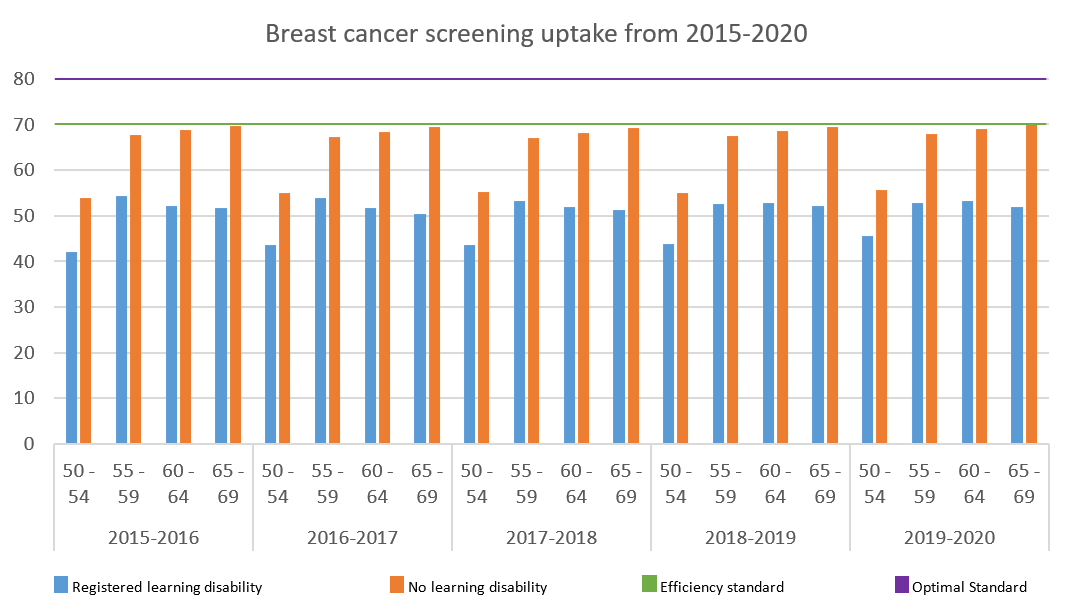 |

| 1. *Colorectal cancer screening uptake* |
| --- |
| 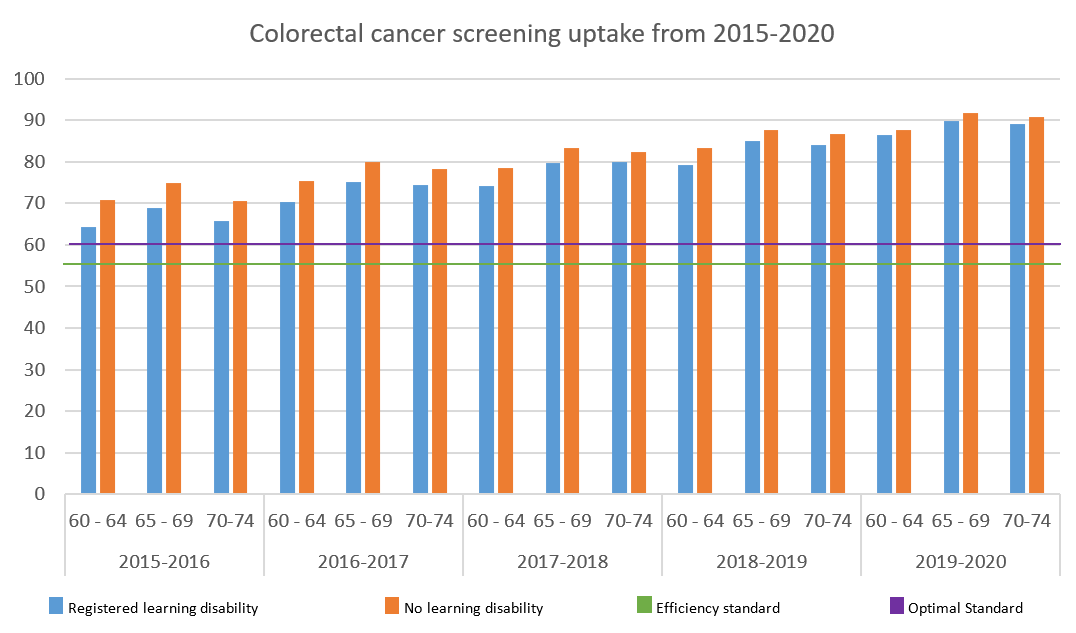 |

Supp 2: Concourse statements

| Source | Statements |
| --- | --- |
| Women with learning disabilities are least likely to attend breast cancer screening – except in Cornwall  Retrieved from - https://phescreening.blog.gov.uk/2017/10/31/women-with-learning-disabilities-are-least-likely-to-attend-breast-cancer-screening-except-in-cornwall/ | 1. Carers and family members can also help women with learning disabilities understand why breast screening is important and attend their appointments 2. Share or read through an easy guide to breast screening or use this breast cancer and breast screening information. Also access leaflets which show you how to check your breasts and order free reminder stickers to put up in the shower 3. In the UK, women with learning disabilities are least likely to attend breast screening 4. Families and carers of women with learning disabilities can play a vital part in helping address this recognised health inequality. 5. Women with learning disability aren’t receiving equal opportunity in health care 6. I have seen easy-read information on breast cancer screening 7. Barriers – letters, fear of the unknown, not understanding the importance of cancer screening, 8. Reasonable adjustments – time, talk and understand the person, sing to help relax, HCP getting their picture taken so the women knows who to expect |
| Making Reasonable Adjustments to Cancer Screening  Retrieved from - https://www.ndti.org.uk/uploads/files/Cancer_screening.pdf | 1. I would know the symptoms of breast cancer 2. I would know the symptoms of cervical cancer 3. I would know who to speak to if I was worried about my breasts 4. The need to increase the knowledge and confidence of carers to support women to attend breast screening / take care of their breasts. 5. An invite letter alone is not enough. |
| Confidential Inquiry into premature deaths of people with learning disabilities (CIPOLD)  Retrieved from - https://www.bristol.ac.uk/media-library/sites/cipold/migrated/documents/fullfinalreport.pdf | 1. Evidence of some difficulties for women with learning disabilities accessing cervical screening, because of presumptions being made about their sexual histories or current sexual activity. 2. Some women were excluded from the screening even though nothing was known about their past history, and 1 care home appeared to have a ‘blanket’ policy of not sending women for screening. |
| Cervical screening for people with a learning disability  Retrieved from - https://www.jostrust.org.uk/about-cervical-cancer/cervical-screening-smear-test-and-abnormal-cells/women-learning-disabilities" | 1. At the moment very few WwLD have a test, far less than other women 2. This might be because some carers, parents or health professionals don't think WwLD need one or because they don't know that they are having sex 3. They may also be too embarrassed to talk about it 4. It is very important that WwLD are given the option to attend their smear test the same as all other women are.   Video:   1. Not reading letters and ignore letters. ‘take no notice of the letters’ 2. Carer opens letter 3. To look after ourselves 4. “A bit uncomfortable but its got to be done” 5. “Its not that bad” 6. The lady behind the desk didn’t think I needed a smear test 7. I kind of persuaded her to get it done |
| South Tees CCG Breast cancer screening video  Retrieved from - http://www.southteesccg.nhs.uk/cancer-screening-services-females-learning-disabilities/ | 1. ‘don’t know’ – what happens when attending breast screening 2. ‘in case you get stuck in the machine and you are left in the dark and in case they find any lumps’ 3. ‘no’ – do you know what breast screening is 4. ‘yes’ – been for breast screening 5. ‘frightened bit nervous and then the hospital said you have to have it done and the two ladies to help me with my breasts’ 6. ‘little bits… are they going to find anything and how do you get the screening done’ – worries 7. ‘if they talk to you about how they are going to do it’ 8. ‘have a teddy or something to cuddle’ 9. ‘a bit nervous, unsure, but I wouldn’t come myself but at least I have seen it now’ 10. Come with support – ‘maybe’ |
| Published literature based on research retrieved from literature searching and the completion of a systematic review  Information extracted from results sections and discussions.  (Note **bold text** is the theme extracted from qualitative studies) | |
| Reynolds, Stanistreet and Elton - Women with learning disabilities and access to cervical screening: retrospective cohort study using case control methods  Retrieved from - https://www.ncbi.nlm.nih.gov/pmc/articles/PMC2248570/ | 1. Women with learning disabilities were significantly more likely to be ceased from screening raising concerns about equity of access to screening programmes. 2. Women with learning disabilities made up 66% of the total number of women who had been ceased. |
| Willis, Kennedy & Kilbride - Breast cancer screening in  women with learning  disabilities: current knowledge  and considerations  Retrieved from - https://onlinelibrary.wiley.com/doi/pdf/10.1111/j.1468-3156.2008.00520.x | 1. Up-take of breast cancer screening was lower in women with learning disabilities than women in the general population. However Two British studies have reported higher up-take of breast screening in women with learning disabilities. 2. General practitioners feeling it was inappropriate for these women. 3. Method of referral for breast screening is one barrier in the UK being registered with a general practitioner is used. In the UK many people with learning disabilities are not registered with a general practitioner 4. May result in some women with learning disabilities not receiving an invitation for breast screening. 5. Other barriers for example physical disability, intellectual ability level, ill health of either women with learning disabilities or carer, moving area/into a nursing home, transport and fear of the procedure 6. Doctors and other health professionals need to know women with learning disabilities can get breast cancer 7. Women with learning disabilities may lacking knowledge about breast screening 8. Women identified as having a learning disability were sent adapted letters about screening. 9. Need to improve education of women and carers on breast awareness and up-take of breast screening 10. If women with learning disabilities are unable to attend for breast screening, breast awareness should still be encouraged 11. Greater acceptability of breast screening compared to cervical screening. 12. GP and practice nurses could play a bigger role in breast screening for these women 13. women with learning disabilities should have the opportunity for breast examinations to be conducted – recommendations are made regarding the issues of abuse and who should perform the examination 14. GPs support the notion of screening of equity of access. Concern raised regarding GP attitude towards breast screening due to high the risk factors of breast cancer in women with learning disabilities. 15. Increased collaboration between primary and secondary care providers enables barriers to be broken down. 16. Carers are more receptive to supporting women being breast aware and through breast screening. |
| Stein - Caring for people with learning disability: a survey of general practitioners’ attitudes in Southampton and South-west Hampshire  Retrieved from -https://onlinelibrary.wiley.com/doi/epdf/10.1046/j.1468-3156.2000.00006.x | 1. Some GPs indicated the potential for uncertainty in assessing a history of sexual activity and one of those who answered ‘unsure’ mentioned medico legal difficulties around obtaining informed consent. 2. A small minority suggested that they would take no action to follow up anon-attendance for mammography. |
| Lloyd & Coulson - The role of learning disability nurses in promoting cervical screening uptake in women with intellectual disabilities: A qualitative study  Retrieved from - https://journals.sagepub.com/doi/pdf/10.1177/1744629514528829 | 1. **The psychological impact of cervical screening** - participants frequently referred to the invasive and inherently unpleasant nature of cervical screening and there was a widely held perception that psychological factors impact negatively on many women’s attitudes towards and ability to tolerate the procedure. 2. **The influence of women’s limited health literacy and competing demands on attitudes towards screening** - Many women with intellectual disabilities would discard the screening invitation letter as a result of this. There was also a perception that many women have limited understanding of the nature and implications of the cervical screening test. 3. **The value of the preparation intervention** - Participants’ accounts illustrated that various strategies are employed as part of interventions to prepare women psychologically for screening in order to enhance understanding, increase predictability and minimise anxiety. 4. **The value of the learning disability nurse’s flexible approach.** - The process of preparing women psychologically for cervical screening can be a prolonged journey that may involve multiple screening attempts prior to a successful outcome. 5. **Balancing women’s rights against the potential for distress** – The ethical issues that can arise during the course of supporting women to access cervical screening and emphasised the role of the learning disability nurse in managing these challenges. It was evident that balancing women’s right to access screening against the potential for significant distress, is an important consideration of the learning disability nurse. 6. **The value of the learning disability nurse’s expertise.** - The value of expertise in managing the challenges of supporting women with more complex needs recurred across accounts. The importance of this expertise in relation to implementing an intervention requiring specialist skills due to the complexities involved and ensuring the necessary procedures are adhered to. 7. **Optimising women’s experiences of cervical screening: The importance of knowledge, attitudes and skills**. - The contribution of primary care professionals’ knowledge, attitudes and skills to patient-directed behaviour and the influence of these competencies on women’s experience and the outcome of cervical screening. 8. **The problem of minimal exposure to people with intellectual disabilities** - An issue was primary care professionals’ lack of exposure to people with intellectual disabilities and the inevitable impact of this on the development of knowledge and skills and women’s experiences of cervical screening. 9. **The issue of time pressures** - negative impact of time constraints when describing their experiences of supporting women to access cervical screening 10. **The importance of facilitating reasonable adjustment**s. - When asked what specifically made cervical screening successful or hindered the process when it was not, the fundamental importance of primary care professionals facilitating reasonable adjustments was highlighted. |
| McIlfatrick, Taggart & Truesdale-Kennedy - Supporting women with intellectual disabilities to access breast cancer screening: a healthcare professional perspective  Retrieved from - https://onlinelibrary.wiley.com/doi/epdf/10.1111/j.1365-2354.2010.01221.x | 1. **Knowledge and awareness of breast cancer and breast screening** - It is important for women with a intellectual disability to undergo regular breast screening, primarily for early detection and prevention. Participants felt strongly that women with intellectual disability should have the same rights as other women to access breast screening services. 2. **Role in supporting women with intellectual disability access breast screening services** - Two main roles emerged for health professionals (both in primary care and breast screening services) in relation to supporting women with intellectual disability to access breast screening services. These roles included: health promotion and providing education and support. 3. **Barriers related to personal aspects of WwLD** - women’s cognitive deficits, communication and level of understanding would pose as a barrier to them accessing breast screening services. 4. **Barriers attributed to carers** - the benefits and value of having someone to accompany the women with intellectual disability to their appointment and that the lack of carer support can be a potential barrier. 5. **Practical barriers** - A number of practical barriers, such as transport and timing of the appointment were noted. 6. **Barriers attributed to healthcare professionals** - Healthcare professional attitudes and experience of working with people with intellectual disability were identified as a barrier. 7. **Solutions to women with intellectual disability accessing breast screening services** - The need for increased aware-ness and health promotion education not only for ‘women with intellectual disability’ and their ‘carers’ but also for all healthcare professionals, including what is tradition-ally perceived as ‘community’ and ‘hospital’ staff. |
| Langan, Whitfield & Russell - Paid and unpaid carers: their role in and satisfaction with primary care for people with learning disabilities.  Retrieved from – requested through the British Library | 1. **Promoting health** – ensuring women had been for cervical and breast screening but carers identified that they could find this uncomfortable as well as their sexual health being dismissed. |
| Levi et al - “Women with learning disabilities and carers  experiences and views of cervical screening” A research project undertaken in South Central and South West Edinburgh LHPs and in Lothian  Retrieved from - https://www.choiceforum.org/docs/csmear.pdf | 1. **Being invited to screening –** In receiving their most recent invitation for a smear test. 2. **Information on cervical screening and the smear test** - None of the women interviewed recalled receiving any written information from health professionals in their most recent invitation for a smear test although several women recalled receiving written information from health professionals in their previous smear invitations as their main source of information regarding cervical screening 3. **Knowledge of cervical screening: purpose and process** - There was a variety of ways in which women described the purpose of cervical screening, with women who had not had a smear test appearing slightly less knowledgeable about the purpose of the test and those who had further investigation or treatment appearing particularly well-informed. 4. **Factors that influenced the woman to have a smear test, or not have a smear test** - The involvement of individual women in the decision about whether or not to have a smear varied considerably. Whereas some women were central to the decision, others were largely excluded from it. One woman made an autonomous decision to have a smear test. Several of the women described that they were supported by either their carer or health professional and in some cases by both in deciding whether or not to have a smear test. 5. **Having the smear test -** Different factors influenced how the woman experienced the smear test. The majority of the women interviewed went for their smear test without support and further, when prompted, stressed that they did not want support to attend. Those who requested support to attend went with their respective carers. The health professionals approach to the woman whilst conducting the smear test was critical to the woman’s experience of screening. Confidence and trust in the professional themselves, and in their ability to undertake the test competently were contributory factors to how the woman experienced the smear test. 6. **Smear test result** - Those women who had been for a smear test had all received their results; one woman had contacted her GP surgery by telephone for the receptionist to issue her results, whereas the remainder received their results by letter. One woman had some difficulty recalling how she had received her result and further, a couple of women could not remember what their last smear test result was. One woman recalled that she had told her doctor that she didn’t understand the difference between a positive and negative result. |
| Broughton & Thomson - Women with learning disabilities: risk behaviours and experiences of the cervical smear test  Retrieved from - https://onlinelibrary.wiley.com/doi/epdf/10.1046/j.1365-2648.2000.t01-1-01555.x | **Themes from women’s' experiences**   1. Women expressed some anxiety about the test. 2. Women who had not received the test felt the most anxiety. 3. Concerned over pain and the nature of the procedure 4. Women who had the test were more likely to make reference to pain experienced as opposed to anxiety about the test Difficulties experienced as a result of feeling tense at the time of under-going the test. 5. Difficulties arose during the process of collecting a cervical smear test 6. Most of the women had a female clinician carry out the test. The women identified that they wanted a female clinician carry out the test 7. Benefits of taking someone along with them to the surgery. Others referred to the support given by medical and nursing staff   **Themes from carers interviews**   1. Carers who had accompanied women for a cervical smear test recognized the need for health centre staff to have an understanding of the specialist needs of the women with learning disabilities 2. The importance of adequate communication and explanation about the cervical smear test. This included getting the message across using visual aids, as well as attention to the type of language used 3. Not considered cervical screening to be an issue for the women, due to assumptions about sexual inactivity, according to medical professionals. 4. The importance of preparation work with the women with learning disabilities. |
| Wood & Douglas - Cervical screening for women with learning disability: current practice and attitudes within primary care in Edinburgh.  Retrieved from - https://onlinelibrary.wiley.com/doi/epdf/10.1111/j.1468-3156.2007.00440.x | 1. **Communication issues** - communication difficulties as the main challenge inherent in providing care to patients with learning disability. The particularly complex issues involved in explaining screening as opposed to therapeutic procedures, the need to enquire about sexual experience, and literacy issues in relation to invitation letters were seen as specific communication issues that added to the general challenge when providing cervical screening to women with learning disability. 2. **Joint working between primary care and Community Learning Disability Teams** – better joint working between primary care staff and Community Learning Disability Team nurses would help to improve the quality of cervical screening for women with learning disability. 3. **Excluding women with learning disability from cervical screening** - women would always be invited for screening, however if after discussion screening was deemed inappropriate for an individual at that time they would not proceed. 4. **Issuing invitations for cervical screening** - The process of issuing the same invitations to all women was preferred as it was seen as equitable, simpler for the practice, and avoided potentially insulting people by sending simplified letters or information leaflets to selected recipients. There was recognition, however, that it relied on carers reading the mail of some patient. 5. **Preparation and smear taking** - the importance of offering preparatory consultations prior to taking a smear. 6. **Care of women unable to give informed consent to screening** – HCP would never perform cervical screening on a woman who was unable to give informed consent and did not appear happy to have a smear taken. Regarding women who were unable to give informed consent but appeared happy to have a smear taken, most interviewees would just go ahead and take the smear, preferably in the presence of the woman’s carer as a chaperone. |
| Truesdale-Kennedy, Taggart & McIlfatrick - Breast cancer knowledge among women with intellectual disabilities and their experiences of receiving breast mammography  Retrieved from - https://onlinelibrary.wiley.com/doi/epdf/10.1111/j.1365-2648.2010.05595.x | 1. **Knowledge** - The women had heard of the term ‘cancer’ and that there were many forms of cancer such as bowel, stomach, lung and skin cancer. However, when asked to describe what cancer was, there was limited knowledge. 2. **Signs and symptoms** - Limited knowledge around the signs and symptoms of breast cancer was also evident with only a small number of women able to name ‘a lump’, ‘spots’ and ‘a red area’. 3. **Risk and protective factors** - The main causative risk factors reported by the women to be associated with breast cancer were related to lifestyle; such as ‘smoking’, ‘diet’, ‘lack of exercise’ and ‘drinking’. In relation to the protective factors for breast cancer, again, much probing was required for the women to identify those same factors, which are aforementioned a risk factors namely an overall improvement in lifestyle. 4. **Source of awareness** - When asked where they had heard about breast awareness and screening it was evident that for most women, the topic was only brought up on receipt of an invitation for breast mammography or from watching a hospital drama on the television. 5. **Women’s experiences of breast mammography: Lack of understanding** - A lack of understanding about the breast screening process was associated with increased stress and anxiety. 6. **Women’s experiences of breast mammography: Fear, pain and discomfort** - ‘fear’ was often attributed to the unfamiliar surroundings and in particular the mammography equipment. 7. **Women’s experiences of breast mammography: Positive experience** - Despite the fact that the women had described their initial fears and anxieties prior to the mammogram and feelings of pain and discomfort during it, once the women had completed the screening process they said that the procedure was not as bad as they had thought. 8. **Women’s experiences of breast mammography: Support from nurses and carers** - the friendliness of the staff at the breast screening unit describing them as ‘chirpy’ and ‘friendly’ and ‘kind’, which helped to alleviate some of their initial fears and anxieties. 9. **Perceived barriers to attendance** - some women with an intellectual disability might refuse to attend breast screening. Two main barriers were identified by women for nonattendance for breast screening; these were ‘fear’ and ‘embarrassment’. 10. **Perceived solutions to barriers** – Solutions for women accessing breast screening services was centred on support both informational and emotional. Which included user friendly leaflets or posters would be helpful in explaining breast awareness, breast screening and prevention albeit the information should be accessible with the writing ‘big’ and ‘easy to understand’. |
| Taggart, Truesdale-Kennedy & McIlfatrick - The role of community nurses and residential staff in supporting women with intellectual disability to access breast screening services  Retrieved from - https://onlinelibrary.wiley.com/doi/epdf/10.1111/j.1365-2788.2010.01345.x | 1. **Importance of breast screening** – consensus for the women to undertake regular breast screening, with the participants highlighting the importance of early detection and the prevention of breast cancer from developing into a terminal illness. Women ‘were entitled to the same services... ...rights’ 2. **Risk factors for breast cancer** - nurses and residential staffs’ knowledge of the risk factors associated with breast cancer, the majority of participants were informed. ‘Family history’ and ‘genetic inheritance’ were clearly identified by many as a leading cause of breast cancer; and also other cancers. 3. **Signs/symptoms of breast cancer** - being aware of some of the signs/symptoms (i.e. changes in breast shape/size/colour, presence of a lump, discharge, pain, rash/inflammation). However, the residential staff reported fewer signs/symptoms than the CIDN. Moreover, neither group identified swelling under the armpit and collarbone, pain in part of the armpit, additional changes in the nipple such as shape, position or rash 4. **Health education and health promotion** - supporting the women to attend their GP, practice nurse and if needed the breast screening clinics. This included ‘providing user-friendly literature’ on how to look after your breasts, supporting the women to self-examine and report any abnormalities 5. **Invitation letter and use of friendly literature** - The main point of access was from the invitation letter forwarded by breast screening unit: with both CIDN and residential staff supporting the women to read the letter. Where available user-friendly literature was also used to educate these women to become self-aware and to explain the process of the breast screening clinic 6. **Recalling upon positive past experiences** - The participants reported that for some women attending the breast screening clinic the experience was reported to be positive, as clear information was given in an accessible format which made the procedure less frightening and uncomplicated. 7. **Lack of knowledge and understanding** - As a result of the women’s poor literacy skills, limited communication skills and level of understanding, they would have limited knowledge about breast cancer and the need for regularly undertaking self-examinations and going for breast screening. 8. **Negative emotions, attitudes and physical barriers** - ‘privacy’ (i.e. fear of undressing), ‘fear and anxiety’ (i.e. the procedure itself and also the potential outcome if signs discovered to be genuine), ‘discomfort and pain’, ‘practical considerations’ (i.e. appointment clashes with day-time activities, physical distance of hospital from home, cost of transport, wheelchair access, car parking) and ‘additional health problems’ (i.e. physical disabilities, coexisting mental health conditions). Staff resources within residential settings were also raised. 9. **Lack of health promotion and education** - Women and also their FCs and/or residential staff, were only made aware of the woman’s appointment for breast screening after receiving an invitation letter from the breast screening unit. Some of the participants highlighted that there was a lack of ‘importance’ and also a dearth of health promotion (i.e. ‘advertising’) and health education (i.e. ‘literature’) in relation to breast screening for women. 10. **Lack of importance given to the need for breast examination and breast screening** – some FCs they made the decision not to inform their daughter/sibling about the breast screening appointment. FCs ‘didn’t perceive the appointment as a priority’, ‘it was a taboo subject that they (FCs) did not want their loved ones (woman with an ID) to be aware of’ or ‘it wasn’t regarded as appropriate for their family member’. 11. **Development of health education material** - consensus among the participants to develop a range of health education material that was ‘user friendly’, using ‘pictures, symbols, signs’ (i.e. ‘Makaton’, ‘Widget’) and simplified words. Information leaflets, booklets and videos/DVDs were highlighted as the appropriate mediums for this information that should be placed in a wide range of facilities such as residential facilities, health centres, day centres and also in the breast screening clinics 12. **Development of health promotional activities** - Educating the women with ID to ‘self-examine’, which focussed upon the ability of the women and whether there were associated physical disabilities and other related health conditions. 13. **Training for all -** that women with ID, FCs, day-care staff, residential staff, CIDN, GPs, practice nurses, breast screening nurses, radiographers and oncology staff required education and training on working with women with ID. |
| Willis - Inconsistencies in the roles of family- and paid- carers in monitoring health issues in people with learning disabilities: some implications for the integration of health and social care  Retrieved from - https://onlinelibrary.wiley.com/doi/epdf/10.1111/bld.12082 | 1. **‘Care within boundaries’** explored how the carers interpreted their role and the influence they had on the health of the individual with learning disabilities whom they supported. The common element shared by the carers was that they had undefined roles in relation to overall responsibility for the health of the person they supported. 2. **‘Doing the best we can’,** examined how the carers attempted to monitor health and the challenges that this presented. All the carers acknowledged the difficulty that people with learning disabilities have in keeping healthy due to their limited insight into their health. Consequently, all carers said that they undertook some form of surveillance of their client’s health, including those who said that health was not their remit. 3. **‘The problem is…’,** reflected the difficulties of explaining health interventions to clients. A generic difficulty mentioned by all the carers was in ensuring people with learning disabilities understand what was being said. |
| Willis, Kilbride, Hornsburgh & Kennedy - Paid- and family-carers’ views on supporting women with intellectual disability through breast screening  Retrieved from –  https://www.onlinelibrary.wiley.com/doi/epdf/10.1111/ ecc.12245 | 1. **‘Keeping an eye on things’** examined how the carers tried to ensure that the women kept their breasts healthy. The majority of carers acknowledged that some women with intellectual disabilities would not be able to undertake breast checks themselves. 2. The difficulties of explaining breast cancer screening to the women is represented by the second theme, **‘It could be broccoli’**. All the carers reinforced the need for reassurance about the procedure when discussing breast screening with the women. 3. **‘A few more difficulties’,** explored the experience of going for breast screening. Irrespective of who made the decision to participate, all the carers believed that women with intellectual disabilities should be offered breast screening. |
| Willis - What influences women with intellectual disabilities to attend breast screening? Experiences of women who have and have not participated  Retrieved from - https://onlinelibrary.wiley.com/doi/epdf/10.1111/bld.12158 | 1. **‘Keeping myself healthy’** - this theme explored how the women kept themselves and their breasts healthy. 2. **‘Orange squeezer’**- explored the women’s experience of participating in breast screening and identified the influences the women were exposed to when deciding whether to attend for breast screening. |

Supp 4: Concourse to Q set statements

| Statement | Q-sort statements | Concourse statement |
| --- | --- | --- |
| 1 | Women with learning disabilities know what lady bit cancer is | 32, 98 |
| 2 | Women with learning disabilities know what boob cancer is | 30, 29, 45, 47, 67, 98, 114 |
| 3 | Women with learning disabilities need their carers and family to explain what cancer screening is | 4, 26, 47, 54, 70, 73, 111 |
| 4 | Women with learning disabilities do not attend cancer screening because they are scared | 7, 24, 28, 31, 35, 43, 57, 59, 61, 81, 82, 84, 102. 103, 104, 106, 113, 115, 125 |
| 5 | Women with learning disabilities are more likely to be stopped from going to screening by doctors | 15, 25, 40, 44, 55, 52, 56, 63, 73, 94 |
| 6 | Women with learning disabilities are told about cancer screening by their doctor | 40, 41, 44, 50, 58, 63, 64, 72, 73 |
| 7 | Women with learning disabilities do not always open letters so do not know about their appointment | 7, 13, 20, 21, 42, 46, 58, 69, 75, 76, 92, 95, 112 |
| 8 | Women with learning disabilities need to know the symptoms of cancer | 9, 10, 73, 99, 100, 110, 127 |
| 9 | Women with learning disabilities speak to paid carers more than their family about their health | 11, 33, 50, 53, 60, 76, 79, 89, 91, 92, 96, 101, 112, 121, 122, 125 |
| 10 | Women with learning disabilities do not attend cancer screening because they are not told how important it is | 7, 17, 41, 47, 55, 58, 72, 79 |
| 11 | Women with learning disabilities do not need to attend lady bits screening if they have not had sex | 14, 17, 72, 74, 90, 92, 94 |
| 12 | Women with learning disabilities are more likely to go for boob cancer screening, than lady bits cancer screening | 3, 16, 39, 49 |
| 13 | Women with learning disabilities know how to check their boobs | 2 47, 48, 51, 67, 114, 119, 122, 124, 127 |
| 14 | Women with learning disabilities are helped to attend screening by talking to their doctor and nurses about what happens | 33, 44, 50, 72, 79, 91, 101 |
| 15 | Women with learning disabilities are asked about what would make it easier for them go to cancer screening | 2, 6, 43, 60, 66, 71, 79, 87, 88, 91, 115 |
| 16 | Women with learning disabilities are given the same treatment as other women | 5, 16, 19, 44, 51, 52, 56, 61, 67, 126 |
| 17 | Women with learning disabilities are not told about cancer screening because their carers and family are embarrassed | 1, 12, 17, 18, 70, 91 |
| 18 | Women with learning disabilities are told everything about the screening process, from beginning to end | 27, 29, 30, 33, 35, 45, 76, 77, 80, 96 |
| 19 | Women with learning disabilities are supported to make their own decisions about going to screening | 26, 31, 43, 70, 94, 97, 126 |
| 20 | Women with learning disabilities are given enough time to decide if they want to be screened for cancer | 8, 31, 65, 78 |
| 21 | Women with learning disabilities know about what will happen in the appointment before they go | 27, 28, 29, 32, 68, 45, 67, 76, 116, 123, 125 |
| 22 | Women with learning disabilities are more likely to attend screening if they hear good stories from other people | 104, 105, 113, 125 |
| 23 | Women with learning disabilities are helped to relax during cancer screening | 8, 34, 36, 66, 70, 79 |
| 24 | Women with learning disabilities would like a lady nurse to do their screening | 31, 86 |
| 25 | Women with learning disabilities find screening painful | 23, 74, 83, 84, 85, 103, 115, 128 |
| 26 | Women with learning disabilities need doctors and nurses to know about the extra help they need | 62, 64, 69, 88, 89, 92, 95, 96, 105, 107, 113, 118, 120 |
| 27 | Women with learning disabilities know the reasons for cancer screening | 22, 28, 32, 45, 67, 68, 70, 77, 100, 108, 122, 123 |
| 28 | Women with learning disabilities have carers who make decisions without speaking to them first | 18, 51, 70, 78, 94, 97, 117, 128 |

Supp 4: Inclusion criteria for participants

|  | Inclusion criteria |
| --- | --- |
| Women with learning disabilities | - Women only, - Aged 18 years or over, - Not currently undergoing treatment for cancer, - Can be a cancer survivor meaning participants have no signs of cancer after they have finished their treatment, - With a diagnosis made by the supporting Health Care team of a mild to moderate learning disability, - Be able to understand English in easy-read, and what is required to complete the study and give consent. |
| Family carer or paid care workers | - Any gender, - Aged 18 years or over, - Be able to read and understand English to read the study documentation, - Be able to give consent to take part, - Must support a person with a Learning Disability either as an FC (not paid), or a PCW (someone who gets paid). |

Supp 5: Q grid


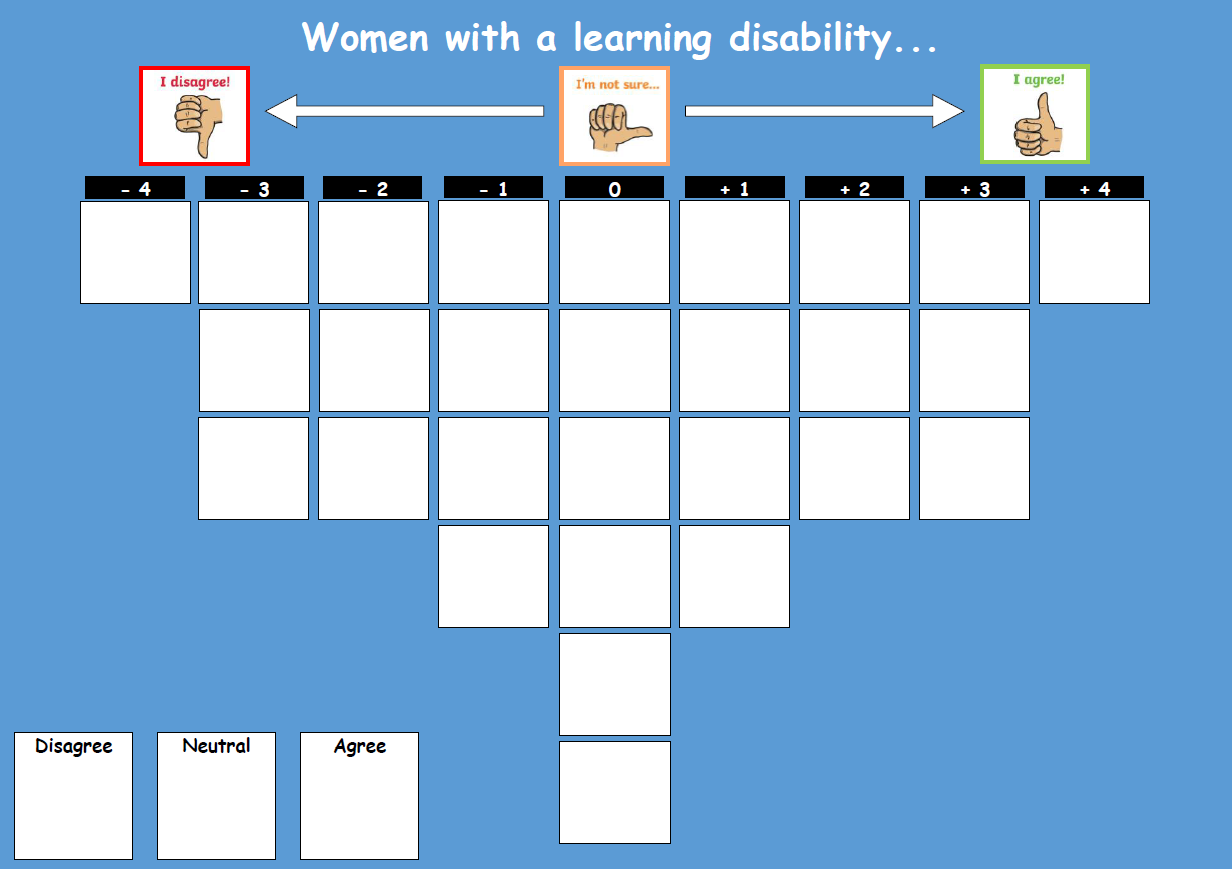


Supp 6: Factor arrays

Factor one


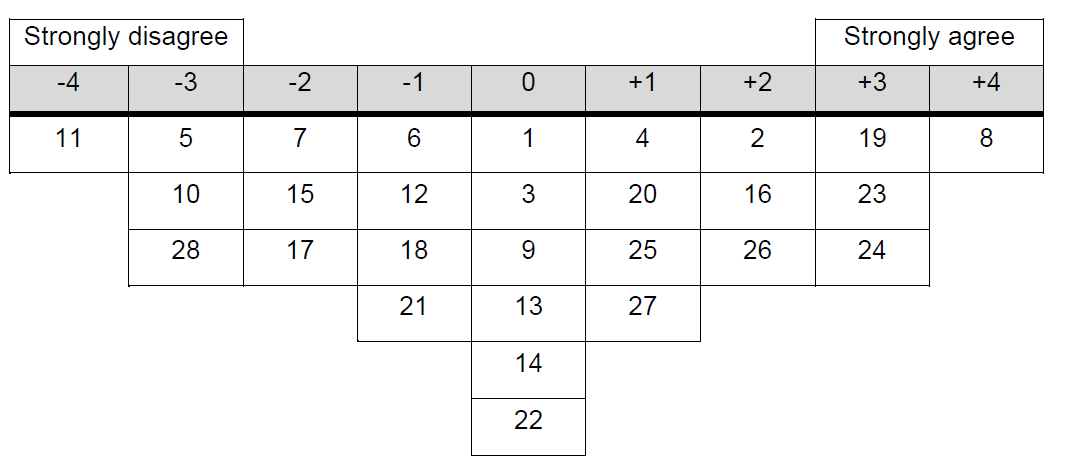


Factor two


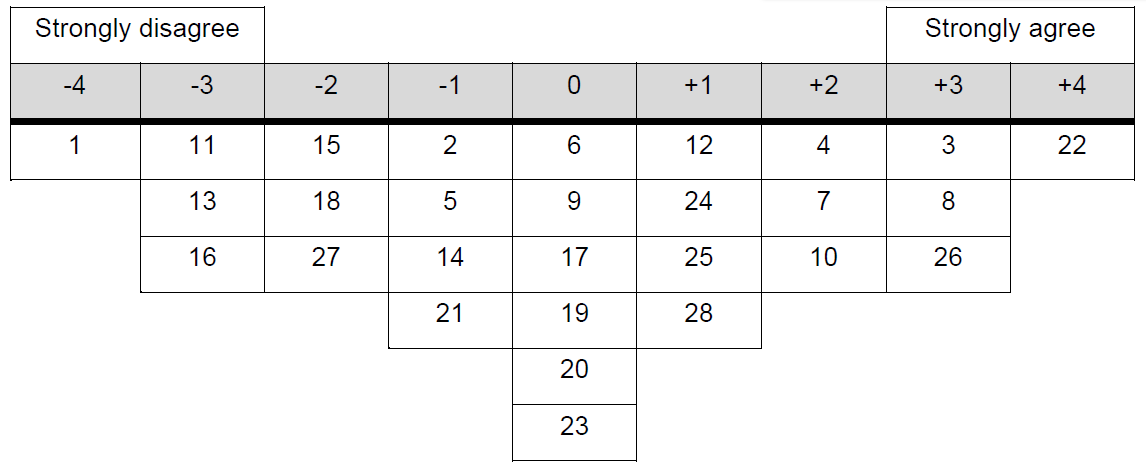

Supplement: Supplementary file 1 — Figure S1: Cancer screening uptake (%) for people with and without learning disabilities from 2015 to 2020 Table S2: Concourse statements Table S3: Concourse to Q‐set statements Table S4: Inclusion criteria for participants Figure S5: Q‐grid Figure S6: Factor arrays [file ECC-31-e13702-s001.docx]
